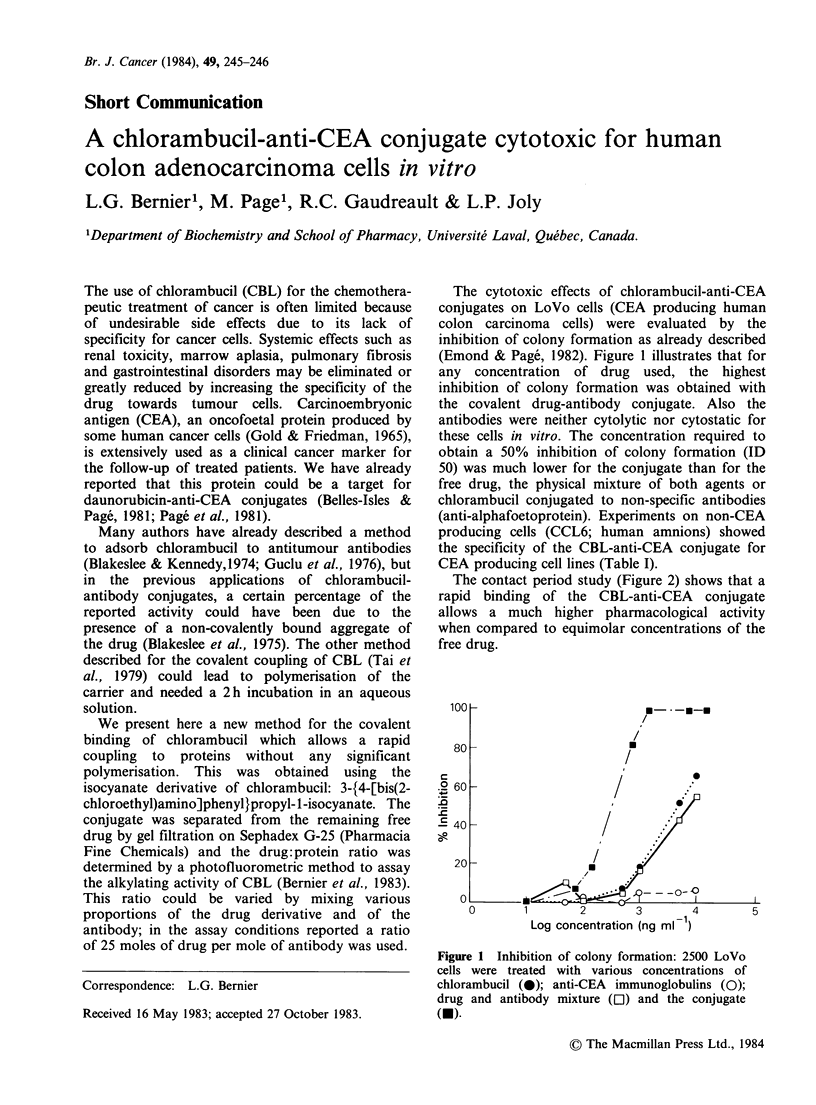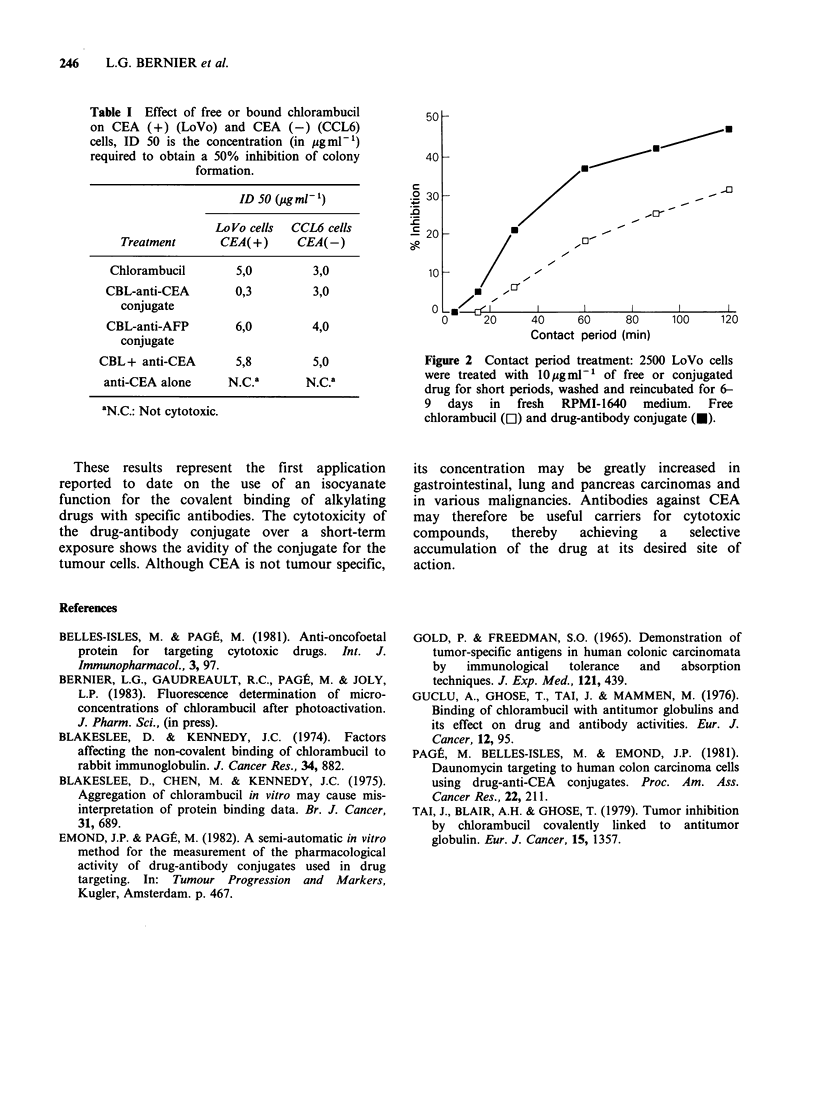# A chlorambucil-anti-CEA conjugate cytotoxic for human colon adenocarcinoma cells in vitro.

**DOI:** 10.1038/bjc.1984.38

**Published:** 1984-02

**Authors:** L. G. Bernier, M. Page, R. C. Gaudreault, L. P. Joly


*Br. J. Cancer* (1984), **49**, 245–246

### Short Communication

### A chlorambucil-anti-CEA conjugate cytotoxic for human colon adenocarcinoma cells *in vitro*

L.G. Bernier[1], M. Page[1], R.C. Gaudreault & L.P. Joly

[1]*Department of Biochemistry and School of Pharmacy, Université Laval, Québec, Canada.*

The use of chlorambucil (CBL) for the chemotherapeutic treatment of cancer is often limited because of undesirable side effects due to its lack of specificity for cancer cells. Systemic effects such as renal toxicity, marrow aplasia, pulmonary fibrosis and gastrointestinal disorders may be eliminated or greatly reduced by increasing the specificity of the drug towards tumour cells. Carcinoembryonic antigen (CEA), an oncofoetal protein produced by some human cancer cells (Gold & Friedman, 1965), is extensively used as a clinical cancer marker for the follow-up of treated patients. We have already reported that this protein could be a target for daunorubicin-anti-CEA conjugates (Belles-Isles & Pagé, 1981; Pagé *et al.*, 1981).

Many authors have already described a method to adsorb chlorambucil to antitumour antibodies (Blakeslee & Kennedy,1974; Guclu *et al.*, 1976), but in the previous applications of chlorambucil-antibody conjugates, a certain percentage of the reported activity could have been due to the presence of a non-covalently bound aggregate of the drug (Blakeslee *et al.*, 1975). The other method described for the covalent coupling of CBL (Tai *et al.*, 1979) could lead to polymerisation of the carrier and needed a 2 h incubation in an aqueous solution.

We present here a new method for the covalent binding of chlorambucil which allows a rapid coupling to proteins without any significant polymerisation. This was obtained using the isocyanate derivative of chlorambucil: 3-{4-[bis(2-chloroethyl)amino]phenyl}propyl-1-isocyanate. The conjugate was separated from the remaining free drug by gel filtration on Sephadex G-25 (Pharmacia Fine Chemicals) and the drug:protein ratio was determined by a photofluorometric method to assay the alkylating activity of CBL (Bernier *et al.*, 1983). This ratio could be varied by mixing various proportions of the drug derivative and of the antibody; in the assay conditions reported a ratio of 25 moles of drug per mole of antibody was used.

Correspondence: L.G. Bernier

Received 16 May 1983; accepted 27 October 1983.

The cytotoxic effects of chlorambucil-anti-CEA conjugates on LoVo cells (CEA producing human colon carcinoma cells) were evaluated by the inhibition of colony formation as already described (Emond & Pagé, 1982). Figure 1 illustrates that for any concentration of drug used, the highest inhibition of colony formation was obtained with the covalent drug-antibody conjugate. Also the antibodies were neither cytolytic nor cytostatic for these cells *in vitro*. The concentration required to obtain a 50% inhibition of colony formation (ID 50) was much lower for the conjugate than for the free drug, the physical mixture of both agents or chlorambucil conjugated to non-specific antibodies (anti-alphafoetoprotein). Experiments on non-CEA producing cells (CCL6; human amnions) showed the specificity of the CBL-anti-CEA conjugate for CEA producing cell lines (Table I).

The contact period study (Figure 2) shows that a rapid binding of the CBL-anti-CEA conjugate allows a much higher pharmacological activity when compared to equimolar concentrations of the free drug.

**Figure 1** Inhibition of colony formation: 2500 LoVo cells were treated with various concentrations of chlorambucil (●); anti-CEA immunoglobulins (○); drug and antibody mixture (□) and the conjugate (■).

© The Macmillan Press Ltd., 1984

**Table I** Effect of free or bound chlorambucil on CEA (+) (LoVo) and CEA (−) (CCL6) cells, ID 50 is the concentration (in $\mu g\,ml^{-1}$) required to obtain a 50% inhibition of colony formation.

| | *ID 50* ($\mu g\,ml^{-1}$) | |
|---|---|---|
| *Treatment* | *LoVo cells CEA*(+) | *CCL6 cells CEA*(−) |
| Chlorambucil | 5,0 | 3,0 |
| CBL-anti-CEA conjugate | 0,3 | 3,0 |
| CBL-anti-AFP conjugate | 6,0 | 4,0 |
| CBL+ anti-CEA | 5,8 | 5,0 |
| anti-CEA alone | N.C.[a] | N.C.[a] |

[a]N.C.: Not cytotoxic.

**Figure 2** Contact period treatment: 2500 LoVo cells were treated with $10\,\mu g\,ml^{-1}$ of free or conjugated drug for short periods, washed and reincubated for 6–9 days in fresh RPMI-1640 medium. Free chlorambucil (□) and drug-antibody conjugate (■).

These results represent the first application reported to date on the use of an isocyanate function for the covalent binding of alkylating drugs with specific antibodies. The cytotoxicity of the drug-antibody conjugate over a short-term exposure shows the avidity of the conjugate for the tumour cells. Although CEA is not tumour specific, its concentration may be greatly increased in gastrointestinal, lung and pancreas carcinomas and in various malignancies. Antibodies against CEA may therefore be useful carriers for cytotoxic compounds, thereby achieving a selective accumulation of the drug at its desired site of action.

**References**

BELLES-ISLES, M. & PAGÉ, M. (1981). Anti-oncofoetal protein for targeting cytotoxic drugs. *Int. J. Immunopharmacol.*, **3**, 97.

BERNIER, L.G., GAUDREAULT, R.C., PAGÉ, M. & JOLY, L.P. (1983). Fluorescence determination of micro-concentrations of chlorambucil after photoactivation. *J. Pharm. Sci.*, (in press).

BLAKESLEE, D. & KENNEDY, J.C. (1974). Factors affecting the non-covalent binding of chlorambucil to rabbit immunoglobulin. *J. Cancer Res.*, **34**, 882.

BLAKESLEE, D., CHEN, M. & KENNEDY, J.C. (1975). Aggregation of chlorambucil *in vitro* may cause mis-interpretation of protein binding data. *Br. J. Cancer*, **31**, 689.

EMOND, J.P. & PAGÉ, M. (1982). A semi-automatic *in vitro* method for the measurement of the pharmacological activity of drug-antibody conjugates used in drug targeting. In: *Tumour Progression and Markers*, Kugler, Amsterdam. p. 467.

GOLD, P. & FREEDMAN, S.O. (1965). Demonstration of tumor-specific antigens in human colonic carcinomata by immunological tolerance and absorption techniques. *J. Exp. Med.*, **121**, 439.

GUCLU, A., GHOSE, T., TAI, J. & MAMMEN, M. (1976). Binding of chlorambucil with antitumor globulins and its effect on drug and antibody activities. *Eur. J. Cancer*, **12**, 95.

PAGÉ, M. BELLES-ISLES, M. & EMOND, J.P. (1981). Daunomycin targeting to human colon carcinoma cells using drug-anti-CEA conjugates. *Proc. Am. Ass. Cancer Res.*, **22**, 211.

TAI, J., BLAIR, A.H. & GHOSE, T. (1979). Tumor inhibition by chlorambucil covalently linked to antitumor globulin. *Eur. J. Cancer*, **15**, 1357.